# Biopolymer and Synthetic Polymer-Based Nanocomposites in Wound Dressing Applications: A Review

**DOI:** 10.3390/polym13121962

**Published:** 2021-06-13

**Authors:** Ravichandran Gobi, Palanisamy Ravichandiran, Ravi Shanker Babu, Dong Jin Yoo

**Affiliations:** 1 Department of Physics, School of Advanced Sciences, Vellore Institute of Technology, Vellore 632014, India; gobirs97@gmail.com; 2 R&D Education Center for Whole Life Cycle R&D of Fuel Cell System, Jeonbuk National University, Jeonju 54896, Korea; ravichandru55@gmail.com; 3 Department of Life Sciences, College of Natural Sciences, Jeonbuk National University, Jeonju 545896, Korea; 4 Department of Energy Storage/Conversion Engineering of Graduate School, Hydrogen and Fuel Cell Research Center, Jeonbuk National University, Jeonju 545896, Korea

**Keywords:** biopolymer, synthetic polymer, biocompatibility, nanomaterials, wound dressing

## Abstract

Biopolymers are materials obtained from a natural origin, such as plants, animals, microorganisms, or other living beings; they are flexible, elastic, or fibrous materials. Polysaccharides and proteins are some of the natural polymers that are widely used in wound dressing applications. In this review paper, we will provide an overview of biopolymers and synthetic polymer-based nanocomposites, which have promising applications in the biomedical research field, such as wound dressings, wound healing, tissue engineering, drug delivery, and medical implants. Since these polymers have intrinsic biocompatibility, low immunogenicity, non-toxicity, and biodegradable properties, they can be used for various clinical applications. The significant advancements in materials research, drug development, nanotechnology, and biotechnology have laid the foundation for changing the biopolymeric structural and functional properties. The properties of biopolymer and synthetic polymers were modified by blending them with nanoparticles, so that these materials can be used as a wound dressing application. Recent wound care issues, such as tissue repairs, scarless healing, and lost tissue integrity, can be treated with blended polymers. Currently, researchers are focusing on metal/metal oxide nanomaterials such as zinc oxide (ZnO), cerium oxide (CeO_2_), silver (Ag), titanium oxide (TiO_2_), iron oxide (Fe_2_O_3_), and other materials (graphene and carbon nanotubes (CNT)). These materials have good antimicrobial properties, as well as action as antibacterial agents. Due to the highly antimicrobial properties of the metal/metal oxide materials, they can be used for wound dressing applications.

## 1. Introduction

**Figure 1:**
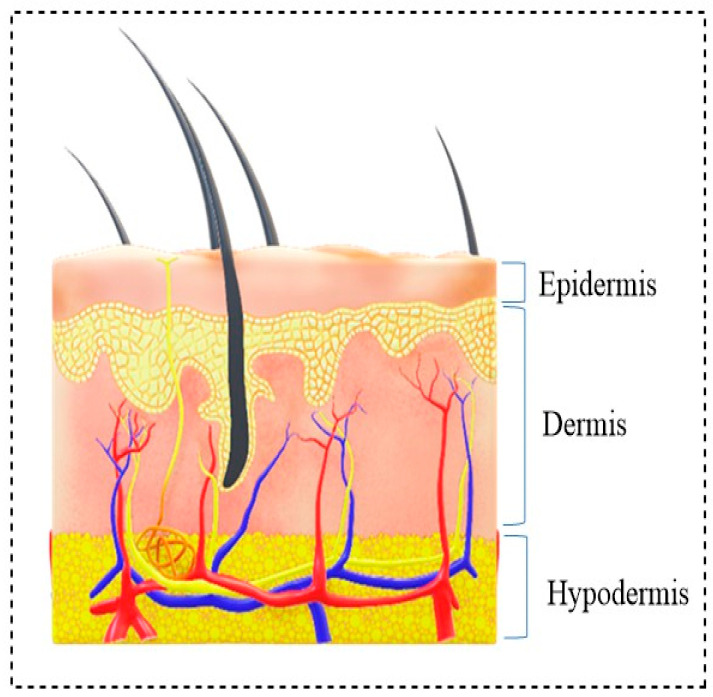
The cross-section view of human skin.

The skin is the largest organ in the human body, and it occupies around one-tenth of the body weight (2 m^2^), providing a natural elasticity [1]. Acting as a barrier, the skin protects our inner organs from foreign bodies such as pathogens, chemicals, and mechanical insults. It also helps to regulate body temperature, prohibits dehydration, and offers support to nerves and blood vessels [2]. The skin prevents extensive body fluid loss, and plays a vital role in thermoregulation and immune defense [3]. Deformations or injuries to the skin’s structure are called wounds. The skin consists of the following three layers: the epidermis, dermis, and hypodermis [4,5], as shown in Figure 1.

**Figure 2:**
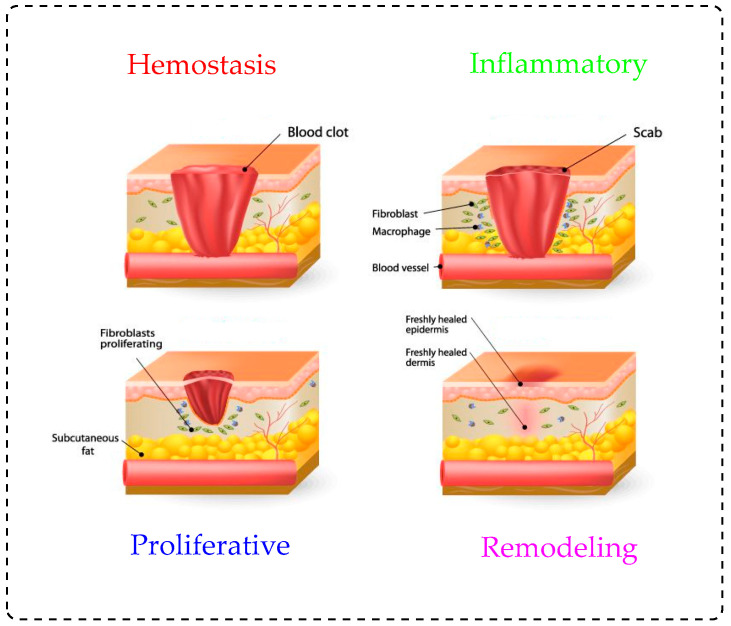
Schematic representation of wound healing stages.

Common injuries to the skin are generated by accidents, burns, chronic wounds, and diseases, which can create a significant healthcare issue. The loss of skin from the body may result in acutely, even patient death [1,6]. Fire-related burns account for 300,000 deaths per annum, and ~25,000 deaths in Europe every year due to bacterial infection, according to the World Health Organization [1,7,8,9,10]. Wounds are categorized into acute and chronic wounds. Injury to the skin that occurs suddenly, due to an accident or surgery, is known as an acute wound. Based on the wound nature in the skin’s epidermal and dermal layers, the time of healing will be around 8–12 weeks [11]. Chronic wounds do not heal within the predicted period, while they are more subject to infections and present significant difficulty in healing [12]. Chronic wounds, such as diabetic ulcers, arterial perfusion, venous insufficiency, and burns, arise in distinct ways [13]. The main function of wound care is to protect the wounds from further damage caused by the accumulation of microorganisms, dehydration, and external agenesis [14]. To maintain skin integrity and homeostasis, wound dressings should facilitate the recovery process by interacting with the wound and releasing bioactive molecules [15]. The growth of tissue has a biological process associated with a specific phenomenon. As seen in Figure 2, the wound healing mechanism is comprised of the following four stages:

1. Hemostasis (immediately after injury);
2. Inflammation (shortly after damage to tissue), during which swelling occurs;
3. Proliferation, where blood vessels and new tissues form;
4. Remodeling (new tissues takes place) provides the proper healing environment [16,17].

Various wound dressing items include non-adherent films, foams, hydrogels, hydrocolloids, hydrofibers, emollients, antimicrobials, charcoal, and composite dressings [18]. The ideal wound dressing should satisfy the following conditions: prevent the wound from physical damage and deter invasion by microorganisms. Moreover, the dressing should be durable and comfortable, create and keep a moist environment, and absorb the wound fluids and exudates; it should also be non-adherent, non-toxic, and a non-irritant, plus stimulate growth factors and be compatible with the healing process [19,20,21]. Dressing materials for various models are now compatible with a variety of wounds, based on the adoption of advanced technologies.

## 2. Biopolymer and Synthetic Polymer-Based Wound Dressing Agents

Natural polymers, also known as biopolymers, are organic compounds produced from living organisms. These polymers contain repeating units of amino acids, nucleotides, esters, or monosaccharides, which are held together by covalent bonds to form polysaccharides, peptides, polyesters, or polyphenols, among others. The source of these biopolymers are animals (chitosan, collagen, and hyaluronic acid), plants (cellulose, natural rubber, and starch), microorganisms (fungi, algae), and bacteria (bacterial cellulose, exopolysaccharides) [22]. Several synthetic polymers, such as PVA and PEG, could be blended with natural polymers to develop new polymeric materials. These newly blended polymeric materials showed enhanced biological properties, specifically wound healing activity. Moreover, the polymeric blends can be strengthened by minerals, which nucleate and grow in a polymeric matrix to produce the appropriate size, shape and distribution of individual crystals, similar to hard tissue. When compared with synthetic materials, these polymers have the advantage, based on their biocompatibility, degradability, recyclability, and lower antigenicity [23].

### 2.1. Biopolymer

A variety of living organisms produce natural polymers such as starch, cellulose, and pectin. Natural polymers are widely used in the regenerative therapeutic sector for burn and wound dressings, due to biodegradability and biocompatibility with the extracellular matrix (ECM). These can be restorative to the damaged tissues and skin regeneration, including the wound healing process. The most widely used natural polymers in biomedical applications are polysaccharides and proteins. This polymer is used in various biomedical applications, such as wound dressings, wound healing, drug delivery, organ transplant, and tissue engineering [24].

Chitin, or (poly-β-(1→4)-*N*-acetyl-d-glucosamine), is a naturally occurring polysaccharide biopolymer, which is usually produced in several living organisms [25]. Natural sources of chitin are the exoskeleton of arthropods, fungi, and the cell walls of yeast. Chitosan is a linear, semi-crystalline polysaccharide composed of (1→4)-2-acetamido-2-deoxy-β-d-glucan (*N*-acetyl d-glucosamine) and (1→4)-2-amino-2-deoxy-β-d-glucan (d-glucosamine) units, a cationic polysaccharide, which is derived from chitin. The cationic nature of chitosan is due to the free amino groups left by the partial elimination of the chitin acetyl groups [26,27]. However, it also presents challenges, particularly for biological applications that need a neutralized environment [28]. Among the polysaccharides, starch is the second most abundant compound accessible in green plants, e.g., corn, rice, potato, and wheat crops. The starch structure is comprised of glucose units linked by glycosidic bonds, and it is semicrystalline. Starch includes two types of molecules, namely, amylose and amylopectin. It has 20 to 25% amylose and 75 to 80% amylopectin by weight, depending on the starch source. A linear polymer of amylose contains glucose molecules that are bound together through (1, 4) glycosidic bonds. Similarly, amylopectin also has (1, 4) glycosidic bonds, but the structure is branched with several side chains of 24–30 glucose monomers, which are held together by (1, 6) glycosidic linkages [29,30]. Cellulose is a linear polysaccharide composed of more than a few hundred to several thousands of β (1→4) linked d-glucose units [31]. Cellulose is the most abundant biopolymer due to its properties, which are essential structural components of marine animals, green plants, bacteria, and algae [32]. Bacterial biosynthetic cellulose is an almost purified type of cellulose, produced using glucose as the typical substratum by strains of the Gram-negative bacterium *Acetobacter xylinum* [33]. Bacterial cellulose membranes have been used for burns as an excellent topical wound dressing. Bacterial cellulose membranes have insufficient intrinsic antibacterial activity; thus, their inability to prevent bacterial disease generally limits the usage in dressings [30,34,35,36,37]. Hyaluronic acid (HA) is a biopolymer of anionic linear polysaccharides, consisting of repeated d-glucuronic acid units and *N*-acetyl-d-glucosamine (1→4) inter glycosidic bonds [38,39,40]. Hyaluronic acid acts as a polyelectrolyte with related cations under physiological conditions, often as a sodium salt; hence, the term sodium hyaluronate. The name was later modified to “hyaluronate” after its salt form or “hyaluronan”, a term used to encompass all forms of the molecule [38]. Hyaluronic acid is a significant constituent of the skin’s extracellular matrix, playing an essential role in cell proliferation and tissue repair [41,42,43]. Alginate is an anionic polysaccharide of a biopolymer derived from the cell walls of brown marine algae, such as *Laminaria* and *Ascophyllum*. Alginate consists of 1, 4-linked β-d-mannuronic acid and β-l-guluronic acid, a linear polysaccharide [44]. Alginic acid and its salts were first used in the form of gels and sponges as wound dressings and treatments [45]. The most influential group focuses on contact with an exuding wound because there is an ion-exchange reaction in the blood serum or wound fluids between Ca^2+^ ions in the membranes and Na^+^ ions [46]. Alginates with a high G block content are widely used in biomedical applications, due to the ease of processing and low immunogenicity [47]. Alginate is used in various biomedical applications, including wound dressings [48,49,50,51].

### 2.2. Synthetic Polymer

Synthetic polymers are chemically synthesized in laboratories, and are also called man-made polymers [52]. Synthetic and natural polymers can produce a new type of material by improving the mechanical properties and biocompatibility associated with single components. They have also been described as biosynthetic or bioartificial polymeric materials [23].

Polyvinyl alcohol (PVA) is a hydrophilic synthetic polymer used extensively in various biomedical fields [53]. PVA is obtained from the presence of a hydroxyl group, which can facilitate the formation of hydrogen bonding [54]. PVA has insufficient elasticity, a thick membrane, and imperfect hydrophilic elements, limiting its utilization alone as a wound dressing polymeric material [16,55,56,57]. Poly(ethylene glycol) (PEG) is a water-soluble synthetic polymer [22]. Growth variables, such as macromers of PEG and epidermal growth factor (EGF), have a strong attraction and can be linked together. It can be applied directly to the site of the wound [58]. By blending the polymer with chitosan and PLGA, properties such as mechanical, thermal, and PEG crystallinity are stabilized. The PEG-based dressing is used to treat diabetic wounds. These dressings facilitate wound healing by initiating the growth and proliferation of skin cells, and collagen deposition [59]. Poly (methacrylic acid) (PMAA) is a synthetic polymer made from the methacrylic acid monomer. PMAA is a hydrophilic and biocompatible substance appropriate for biomedical applications, so bioactive materials, such as drugs or inorganic nanoparticles, can be further combined with a carboxylic acid group [60,61]. This material also has a high swelling capacity in water, and can change distinctly upon variations in the solution’s pH and ionic strength [62,63]. Polyvinylpyrrolidone (PVP) is a water-soluble synthetic linear polymer made from the monomer *N*-vinylpyrrolidone. PVP can interact with a variety of synthetic polymers [64,65]. Polycaprolactone (PCL) is a synthetic polymer, with hydrophobic and semi-crystalline properties [66]. Hence, the crystallization and the morphologic properties of PCL are significant [67]. PCL has been blended or copolymerized with other polymers to improve its mechanical properties [68]. Poly (lactic acid) (PLA) is one of the most extensively used synthetic polymers in biomedical areas. There are several distinct types of polylactide with the chiral structure of lactic acid; poly-L-lactide (PLLA) is the result of L-polymerization, which is referred to as L-lactide [69,70]. The surface of PLA is strongly hydrophobic, but to improve its biocompatibility, various surface and bulk modifications have been shown to enhance the hydrophilicity [71]. Polyurethane (PU) is a thermoplastic copolymer of urethane groups, formed by a polymerization reaction by mixing diol and diisocyanate units. In rare cases, non-biodegradable and non-toxic polyurethane elastomers have been used in wound dressings to cover the burned/injured area of the patients as an external film [29]. Polyurethane is often used in wound dressings because of its good barrier properties and oxygen permeability [72,73]. A summary of biopolymers and synthetic polymers, and their chemical structures, properties and biomedical applications are presented in Table 1.

## 3. Polymeric Nanocomposites in Wound Dressing

Nanotechnology is an emerging field of research that has been used to resolve numerous biological concerns, both therapeutic and diagnostic. Recently, nanotechnology has been broadly studied for use in multiple diseases, such as diabetes, wound healing, cancer, and cardiovascular diseases [74]. Fabricated nanoparticles, developed in the diameter range of less than 100 nm, show limited physicochemical characteristics, due to the large surface-to-volume ratio and increased reactivity [75]. Specifically, metal oxide nanoparticles (MONPs) possess some advantages, such as the following: simple preparation processes and high stability; easy engineering to the desired shape, size, and porosity; easy incorporation into hydrophilic and hydrophobic systems; no swelling variations; and easy functionalization by various molecules, due to the negative charge of the surface, which suggest an encouraging tool for biomedical applications [76]. Healable nanomaterials, in particular, result from nano-scaled material and the nanomaterial’s intrinsic properties as carriers for the distribution of therapeutic agents. The results and wound healing potential of different nanomaterials vary, often based on their physicochemical properties [77,78,79]. Nanomaterials create a different path for wound-healing products, due to their specific properties. Nanomaterials can change each stage of wound healing as they have anti-inflammatory, antibacterial, proangiogenic and proliferative properties. Nanomaterials can also alter the degree of regulation of different essential proteins and signal molecules, which improves wound healing. Thus, both micro and nanoscale nanomaterials or a mixture of materials can become favorable to solve most wound care management challenges [78]. The main classes of nanomaterials used for wound treatment are nanoparticles, nanocomposites, scaffolds, and coatings [80].

### 3.1. Zinc Oxide Embedded in Wound Dressings

Rakhshaei et al. [81] prepared (ZnO-MCM-41) zinc oxide impregnated mesoporous silica carboxymethyl cellulose (CMC) hydrogel as a nano-drug carrier. To prevent the cytotoxicity of usual crosslinkers, citric acid was used as a crosslinker. The swelling and erosion experiments indicate that, within the first hours of the test, the CMC/ZnO nanocomposite hydrogel disintegrated. The use of MCM-41 as a substrate for zinc oxide nanoparticles, and CMC/ZnO-MCM-41, exhibited a significant increase in properties of corrosion (53%), tensile strength (12%), swelling (100%), and gas permeability (500%). After 24 h of release, the microbial assay exhibited a potent antibacterial effect in TC overloaded CMC/ZnO-MCM-41. In adipose tissue-derived stem cells (ADSCs), cytocompatibility of the nanocomposite hydrogel films was investigated, and the findings demonstrated CMC/ZnO-MCM-41 cytocompatibility. For wound dressing and healing activity, these properties may be useful.

Khorasani et al. [82] prepared zinc oxide nanoparticles (ZnO) that were incorporated into heparinized polyvinyl alcohol/chitosan hydrogels and used as wound dressing materials. The hydrogel was prepared by the freeze-thaw method, with two and four freeze–thaw cycles. WVTR in the PVA/CS/ZnO/heparin hydrogel ranges from 2200 to 4550 g/m^2^/day. The cell viability analysis of PVA/CS/nano zinc oxide hydrogels was performed on mouse fibroblast cell viability (L-929). After 24 and 48 h, all of the samples showed cell viability values greater than 70% and 80%, respectively. Adding nano zinc oxide into the hydrogels against *E. coli* and *S. aureus* pathogens, the antibacterial impact was over 70%. Compared with the sample without ZnO, this value was less than 60%. PVA/CS/ZnO/heparin hydrogel nanocomposites efficiently protect the wound surface from dehydration, exudate formation, and bacteria growth, thus increasing the rate of the wound healing process.

**Figure 3:**
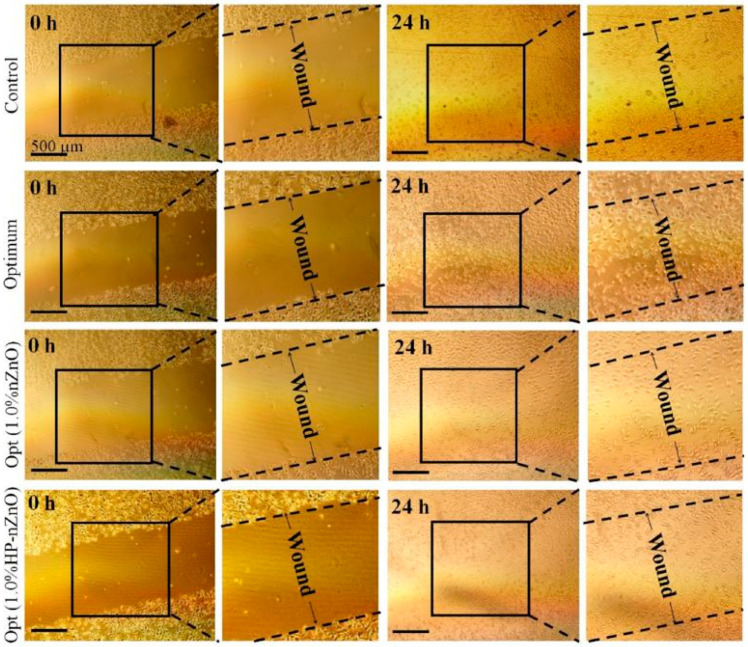
The scratch assay was conducted on prepared wound dressings materials (1% nZnO and 1% HP-nZnO). Reprinted with permission from [83], copyright 2019 Elsevier.

Joorabloo et al. [83] prepared heparinized zinc oxide nanoparticles/poly(vinyl alcohol)/carboxymethyl cellulose processes by the freeze–thaw method. An in vitro cell viability assay was performed on mouse fibroblast cells L-929 and human dermal fibroblasts (HDF) cells. A heparinized nanoparticle sample was contained the antibacterial study by over 70% against *E. coli* and *S. aureus* pathogens. Different ratios of nanoparticles and the sample containing heparinized nanoparticles exhibited non-toxicity after 24 and 48 h. Cell viability was between 72% and 83% after 24 h (compared to a control sample equal to 100%) and significantly improved after 48 h in all samples. The largest zone was examined around the sample consisting of 1 wt.% nZnO and 1 wt.% HP-nZnO. Fabricated dressings were evaluated to have excellent WVTR and DSR (degree of swelling ratio), biocompatibility, and mechanical properties, as well as an excellent ability to heal and protect the wound from bacterial infections. After 24 h of creating artificial wounds, the extract obtained from all samples could heal the wounds properly and nearly filled the wound area (Figure 3), an indication of the fact that the present system could induce cell migration to the trauma region. From the above results, we can conclude that the wound dressing system developed in this particular study is suitable for wound healing in a timely and convenient manner.

Rao et al. [84] prepared hyaluronic acid (HA) and zinc oxide (ZnO) nanocomposite hydrogels by the process of one-pot synthesis. ZnO nanobelt formation supports the HA hydrogel network structure, with the 1,4-butanediol diglycidyl ether (BDDE) as a crosslinker. Human skin fibroblasts were treated with the hydrogels. HA–ZnO NCHs, in comparison, have better biodegradability and swelling. The HA–ZnO nanocomposite hydrogel showed high hemocompatibility, which improved antibacterial effects and blood clotting. In comparison, CCD986sk cells cell proliferation and adhesion to HA–ZnO nanocomposite hydrogel are similar to that of the hyaluronic acid hydrogels. Antibacterial studies against *S. aureus* and *E. coli* pathogens show that they produce a good antibacterial effect. Therefore, future wound dressing applications could have ZnO nanobelt-like structures in hyaluronic acid hydrogels.

Wahab et al. [85] prepared a polyurethane (PU) nanofiber-based nanocomposite, containing silver (Ag)/zinc oxide (Zn) composite nanoparticles, proposed for antibacterial wound dressing applications (PUZnAg). The sample of PUZnAg2 revealed 0% viable bacterial cells. The composite nanofiber’s antibacterial effect was evaluated against *S. aureus*, *E. coli*, and *B. subtilis* pathogens. The PUZnAg2 sample, containing 8% of ZnAg by weight, showed 100% bacterial effects against all three test pathogens. The PUZnAg composite nanofibers strongly prohibit bacterial growth for up to 72 h. PUZnAg composite nanofibers were synthesized for protracted antibacterial wound dressing applications.

Majumder et al. [86] prepared a dressing for biomimic composite wounds by hydrogel grafting of silk fibroin fabric. The main layer of silk woven cloth and an outer layer of hydrogel poly (acrylic acid-co acrylamide) coated with zinc oxide nanoparticles were developed for a composite wound dressing. Using ammonium persulfate (APS) as an initiator and *N*,*N*′-bismethylacrylamide (MBA) as a crosslinker, the hydrogel coating was integrated into the material by grafting acrylamide (AAm) and acrylic acid (AAc) monomers on the silk fiber. The grafted silk fibroin fabric hydrogel’s swelling potential was optimized by adjusting the initiator’s concentration. These dressings are also sonochemically coated with zinc oxide nanoparticles to impart antimicrobial properties. *E. coli* were studied against the antimicrobial activity of zinc oxide-coated samples. Using L929 fibroblast cells, the cytocompatibility of formulated dressing materials was examined. The studies indicate that the dressing has adequate water vapor permeability, good mechanical properties, and essential antibacterial properties. Hydrogel-coated silk has a WVTR value of 480 g/m^2^/day, making it more suitable for secondary and tertiary wounds. The results from the MTT assay and microscopic studies show that the growth, adherence, and proliferation of L929 fibroblast cells, seeded on zinc oxide nanoparticles on hydrogel-grafted silk fibroin, shows a higher dressing ability than the pure silk fibroin. The above-mentioned zinc oxide nanomaterials functionalized on hydrogel-grafted silk fibroin will be a suitable candidate for wound dressing, and also it can be used as regenerative medicine. In vivo trials and clinical studies showed effective dressings.

### 3.2. Cerium Oxide Embedded in Wound Dressings

Naseri et al. [87] incorporated cerium oxide (CeO_2_) nanoparticles into poly (ε-caprolactone) to improve gelatin films as a potential wound dressing material. The electrospinning process for PCL/gelatin (1:1 (*w*/*w*)) solution, to incorporate 1.5, 3, and 6% (*w*/*v*) of CeO_2_ nanoparticles, was used to prepare the wound dressings. Electrospun films were evaluated in terms of their morphology, contact angle, water absorption ability, tensile strength, water vapor transmission rate, and cellular reaction. The highest cell proliferation occurred with L929 cells in the PCL/gelatin film containing 1.5% (*w*/*v*) CeO_2_ nanoparticles. For in vivo analysis of the full-thickness of excisional wounds in Wistar rats, the film incorporating 1.5% CeO_2_ nanoparticles was used as the ideal dressing. The study showed that after 2 weeks, the wound dressing containing CeO_2_ nanoparticles achieved a substantial closure of almost 100% relative to sterile gauze, which showed approximately 63% wound closure. The findings presented evidence to support the potential applications for effective wound care with the CeO_2_ nanoparticle-containing dressing.

**Figure 4:**
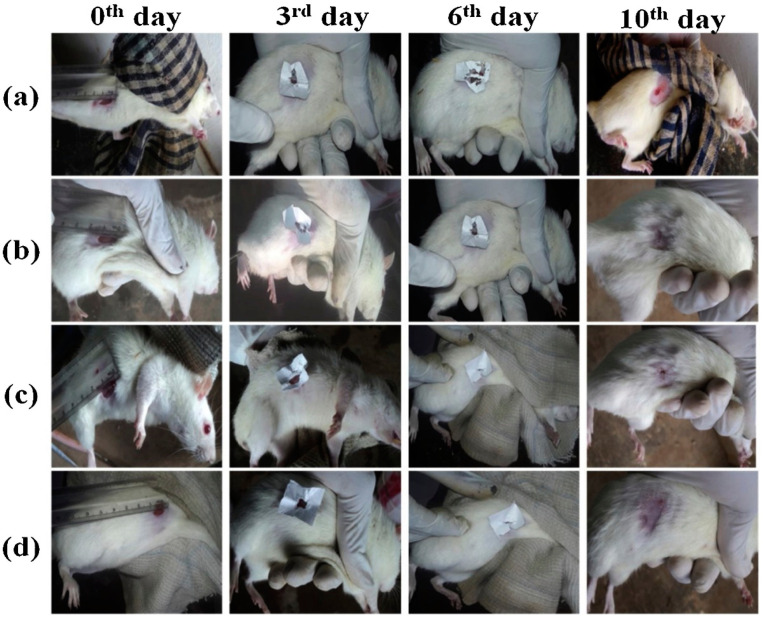
Representative photographs of wound closure on day 0, 3, 6 and 10. (**a**) PEO/GO (**b**) CeO_2_–PEO/GO (**c**) PM oil–PEO/GO, and (**d**) CeO_2_–PM oil–PEO/GO nanofibrous mats. Reprinted with permission from [88], copyright 2019 Elsevier.

Bharathi et al. [88] prepared cerium oxide (CeO_2_) and peppermint oil (PM oil) on polyethylene oxide (PEO)/graphene oxide (GO) polymeric mats by the electrospinning technique. As measured from scanning electron microscope images, the fricated nanofibrous mat’s average fiber diameter is 370 nm. Due to the surface charge of cerium oxide and peppermint oil’s antibacterial properties, the PEO/GO nanofibrous mat containing CeO_2_ and PM oil demonstrated sustained antibacterial action. A disc agar diffusion technique was used to assess the antibacterial movement of the nanofibrous mats against *E. coli* and *S. aureus*. MTT research showed that after incorporating CeO_2_ and PM oil, the nanofibrous mat revealed low cytotoxicity against L929 fibroblast cells. The nanofibrous composite mats wound healing activity can be increased because of the existence of active functional groups in PM oil, and the dual oxidation state of CeO_2_. Histology findings showed that, by facilitating wound contraction, improved collagen deposition, and re-epithelialization, the composite nanofibrous mat exhibited a fast-healing mechanism. The results show that cerium oxide and PM oil composite nanofibrous mats have a low toxicity level against L929 fibroblasts. The wounds treated with the prepared nanofibrous mats showed better wound healing as compared to the control (Figure 4). These antibacterial electrospun nanofibrous mats are used in biomedical applications for next-generation wound dressing materials.

Kalaycıoğlu et al. [89] prepared wound dressing materials made from chitosan (Chit) and cellulose acetate (CelAc) polymer composites with nano cerium oxide (CeO_2_). The polymer composite was made using the solvent casting method. Biopolymer compounds, such as chitosan and cellulose acetate, have biocompatible features. The two polymers can be dissolved in formic acid. Due to the vaporization of formic acid, the sponge-like porous shape formed. The final degradation stage in the TGA for all films occurred at ~350 °C. The films obtained a maximum swelling of ~95% for Chit/CelAc, ~58% for Chit/CelAc/CeO_2_-0.1, and ~31% for Chit/CelAc/CeO_2_-1 in 1 h, according to swelling performance evaluation. The moisture content of the films stored at 25 °C and 65% humidity was gradually decreased with the addition of cerium oxide nanoparticles. The film’s WVTR is found to be between 277 and 566 g/m^2^/day. The water vapor permeability (WVP) of these films varied from 1.45 to 3.73 g·mm/h·m^2^·mmHg. Under visible light, with the Chit/CelAc/CeO_2_-0.1 films, the wound site may be directly observed. The wound is protected from UV light by Chit/CelAc/CeO_2_-0.1 and Chit/CelAc/CeO_2_-1 films. Chit/CelAc and Chit/CelAc/CeO_2_-0.1 have transparency values of 31.52 and 12.72 at 600 nm, respectively. By changing the amount of nano cerium oxide, the antibacterial activity could be controlled. Films have been recommended as ideal wound dressing products.

Kalantari et al. [90] analyzed the CeO_2_ NPs that were developed by a green approach, using the *Zingiber officinale* extract to reduce the toxicity of the compounds in their synthesis. In the PVA/chitosan/CeO_2_ NPs hydrogel with 0 to 1% (wt), 5 nm cerium oxide nanoparticles were synthesized by the freeze–thaw process. Then, PVA/CS hydrogels containing 0.5 and 1% CeO_2_ NPs were effectively developed for wound dressing applications. The hydrogels containing 0.5% CeO_2_ NPs had good antibacterial activity after 12 h, and, compared with the control group, human dermal fibroblast cell viabilities existed for up to 5 days (more than 90%). For the hydrogels of 1 percent, 0.5 percent, and the controls, the average pore size was 0.562 ± 0.32 μm, 0.104 ± 0.03 μm and 0.077 ± 0.02 μm, respectively. The contact angle was reduced by adding 0.5 and 1 wt.% CeO_2_ NPs to the hydrogels. The hydrogels were tested for antimicrobial activity against wound-infecting bacteria *E. coli* and *S. aureus*. These cerium nanoparticles that incorporated chitosan/PVA hydrogels may be a promising candidate for a wound dressing agent, because they can effectively reduce wound infections without the use of antibiotics.

### 3.3. Silver Embedded in Wound Dressings

Augustine et al. [91] prepared fibers using an electrospinning technique to produce polycaprolactone (PCL) membranes, which incorporated biosynthesized AgNPs for wound dressing applications. The diameter of the fibers varied greatly, as the concentration of AgNPs increased from 0.05 to 1 wt.%. The tensile strength, tensile modulus, and elongation at break were far greater for up to 0.5 wt.% of AgNPs content for PCL/Ag nanocomposite membranes. After adding 1 wt.% silver nanoparticles, the intrinsic elastic structure of PCL shifted to a brittle nature. The processed material shows a higher antibacterial assay against *E. coli* and *S. aureus*, which improves the fabricated material’s ability to avoid bacterial infection in wounds protected by this material. These studies showed that PCL/Ag nanocomposite membranes with lower Ag nanoparticle content produce fibers with higher mechanical properties. The inhibitory zone diameters of the PCL membrane with 1 wt.% Ag nanoparticles against *S. aureus* and *E. coli* are 11.6 ± 0.5 and 7.9 ± 0.6, respectively. Bacteria such as *S. aureus* and *E. coli* were inhibited by PCL membranes containing 1 wt.% Ag nanoparticles, which had inhibitory zone diameters of 11.6 ± 0.5 and 7.9 ± 0.6, respectively. Biosynthesized Ag nanoparticles were incorporated with a PCL membrane, which can be used for wound dressing.

Hiep et al. [92] fabricated chitosan/PVA/AgNPs gels with microwave assistance for wound dressing applications. Using irradiation by microwaves, silver ions were reduced to silver nanoparticles, and CS was crosslinked with PVA. The agar diffusion method examined the antimicrobial properties against *S. aureus* and *P. aeruginosa* of the CPA gels. The cell viability of CPA1 and CPA0.5 was approximately 76% and 80%, respectively. The thick scabs were detached on day 11, and the wound sizes of all of the samples were reduced by 80% relative to the original size. Furthermore, the wound that was treated with CPA1 had no scab. In vivo and in vitro studies exhibit that CPA0.5 and CPA1 both have good biocompatibility and protection for wound applications.

Alippilakkotte et al. [93] prepared PLA/Ag nanofibers developed by the electrospinning technique. The colloidal nano silver involved in the device was synthesized from silver nitrate (AgNO_3_). Bitter gourd extract was used as a reducing agent in the diphase agent comprising the polylactide (PLA) matrix capping agent, using the biological reduction process. X-ray photoelectron spectroscopy (XPS) confirmed the capping agent polylactide interaction with a colloidal nano silver. The antibacterial property was observed by the agar disc diffusion method for PLA/Ag nanofibers against *S. aureus* and *E. coli*. PLA/Ag nanofibers demonstrated antimicrobial behavior, and studies indicate that efficiency increased with a lower silver concentration. It was observed that the PLA nanofiber, with 2 wt.% Ag, had a higher tensile property and improved hydrophilicity increased surface wettability. The in vitro study revealed that the processed nanofiber for PLA/Ag (with an HP < 5%) was also cytocompatible with fibroblast cells and did not destroy the growth of cells. The PLA–2Ag nanofiber WVTR was found to be 2237.53 ± 165 g/m^2^ after 24 h, and appeared to provide an excellent moist environment to improve wound healing. These findings confirm that PLA/Ag nanofibers may be used as a wound healer to improve the proliferation and function of fibroblasts and epidermal cells.

Mehrabani et al. [94] synthesized an efficient wound dressing application, a biodegradable and biocompatible silk fibroin/chitin nanocomposite scaffold with an additional silver (Ag) nanoparticle (0.001, 0.01, and 0.1%) content, using the freeze drying method. By increasing the number of AgNPs in the nanocomposite, the porosity level was decreased, and this less porous nanocomposite has an excellent application in wound dressing. Increasing the Ag content also increases the tensile strength of the nanocomposites. For different weight percentages of AgNPs, the corresponding tensile strengths are 0.547 of 0.001%, 0.593 of 0.01%, and 0.650 of 0.1% AgNPs. At 7, 14, and 21 days, the nanocomposite containing 0.001% AgNPs has a strong degradability rate of 32.91%, 54.87%, and 72.19%, respectively. The biocompatibility with high antimicrobial effects, blood clotting, high porosity, good mechanical properties, water uptake, swelling, and biodegradability has been investigated in the nanocomposite scaffolds. In nanocomposite scaffolds, antimicrobial assessments have potent antimicrobial activity, inhibiting the growth of *S. aureus*, *E. coli*, and *C. albicans*. Also, cell viability, cell attachment, and proliferation, with DAPI stain on nHFFF2 cell and MTT assay, have shown the nanocomposite scaffold’s cytocompatible nature. However, a lower concentration of AgNPs (0.01 and 0.001%) positively affects proliferation and cell attachment. All of the results indicate that the prepared nanocomposite scaffolds are good candidates for wound dressing materials.

Sofi et al. [95] fabricated the composite wound dressing nanofibers consisting of polyurethane (PU), lavender oil, and silver nanoparticles, processed by an electrospinning technique. The diameter of the fiber was reduced by the excess of silver (Ag) nanoparticles in the fibers, while the elevated lavender oil content increased the diameter. The contact angle in polyurethane with 5% lavender oil/1% AgNPs is 69.3 ± 1.5°, 10% lavender oil/3% AgNPs is 52 ± 3°, 15% lavender oil/5% AgNPs is 46 ± 2°, and 20% lavender oil/3% AgNPs is 32 ± 2.5°, respectively. The silver nanoparticles and lavender oil enhanced the nanofiber’s hydrophilicity and protected the growth of embryo fibroblasts of in vitro cultured chicken on these fiber dressings. *S. aureus* and *E. coli* were used to analyze the antibacterial activity of nanofiber dressings, yielding 16.2 ± 0.8 and 5.9 ± 0.5 mm inhibition areas, which prove good bactericidal properties of the dressings. Studies on the effectiveness of these dressings on superficial and deep wounds needs further study, particularly in an effective animal model, using a 15% lavender oil and 5% AgNPs blend as the highest concentration. There is a tremendous opportunity for composite nanofiber dressings to be used as useful wound dressings to provide protection against foreign agents and encourage the growth of new tissues.

Li et al. [96] prepared a sequence of silver nanoparticles (AgNPs) and eggshell membrane (ESM) composites (AgNPs/ESM). Using the chemical reduction process, Ag nanoparticles were prepared. In order to find the optimum condition for processing AgNPs/ESM composites, various pH and processing time combinations were tested. The stock solution of AgNPs was diluted 2, 4, 6, 8 or 10 times with water to achieve the optimum nontoxic-level Ag release. The silver composite concentration was 2.41 mg/L, which was below the 3.3 mg/L non-toxic doses. By adding AgNPs, the water contact angles of ESM changed from 105° to 75°, which changed the hydrophobic nature of ESM to a hydrophilic nature, which is important for wound healing. The antibacterial properties of AgNPs/ESM composites against *S. aureus* and *E. coli* were investigated. The surface area of AgNPs/ESM (159.08 m^2^/g) composites is larger when compared with the natural ESM (24.32 m^2^/g), and the appropriate pore size (10.92 nm) enhances the absorption and antibacterial properties. These results clearly portray that the AgNPs/ESM composites could be an effective candidate for developing antimicrobial agents for therapeutic and biomedical applications, such as wound healing.

Nasef et al. [97] used gamma irradiation to prepare a composite crosslinked hydrogel membrane made of polyvinyl alcohol (PVA)- and chitosan (CS)-loaded AgNO_3_, and vitamin E. Copolymer concentration, AgNO_3_ concentration, irradiation dose, vitamin E, and plasticizer with PVA/chitosan membranes were analyzed to study the influence of these parameters on the formation of the hydrogel membrane. The mechanical and thermal properties of the hydrogel composite membranes were also studied to evaluate the applicability as a wound dressing. By increasing the radiation dose and AgNPs ratio, the swelling ratio of the composed hydrogel membranes decreased considerably; this is due to the effect of the incorporated AgNPs to reduce the degree of crosslinking in hydrogel membranes. Also, for the same reason, the swelling ratio of the composite hydrogel membranes decreased significantly as the radiation dose and AgNPs incorporation increased. PVA–CS–Ag composite hydrogel membranes exposed strong antimicrobial pathogens against *S. mutans* compared to other bacterial and fungal microbes, due to the presence of AgNPs in the membranes. The composite membranes of PVA/chitosan/AgNO_3_-Vit.E hydrogel exhibited excellent properties as a wound dressing material.

Ahmed et al. [98] fabricated a PVA and starch composite polymeric membrane as a wound dressing. The filler was graphitic carbon nitride (g-C_3_N_4_), and the antibacterial agent was silver-deposited titania nanoparticles (Ag@TiO_2_ NPs). The hydrogel membranes were prepared under ambient conditions by a solvent casting technique. *E. coli* and *S. aureus* were treated for antibacterial activity. For the PVA/starch/0.14GCN/0.7Ag@TiO_2_ NPs composition, the maximal inhibition zones obtained were 37.33 and 33.25 mm, respectively. The hydrogels showed an excellent swelling capacity against saline, water, and simulated wound fluid, for up to 144 h. Hydrogels can retain up to 90% of a significant volume of moisture. Porosity and oxygen permeability studies have demonstrated membrane breathability, as it is necessary for nutrient and oxygen exchange. The results of porosity and oxygen permeability revealed that the membranes of hydrogels were breathable. Using different mathematical models, drug release was conducted, where the Higuchian model was the best fit. In 7 days, complete healing was reached. The fabricated hydrogel membranes demonstrated faster healing than traditional wound dressings made of cotton gauze. The prepared hydrogel membranes have shown that they can be used as a wound dressing for both partial and full-thickness excision wounds.

**Figure 5:**
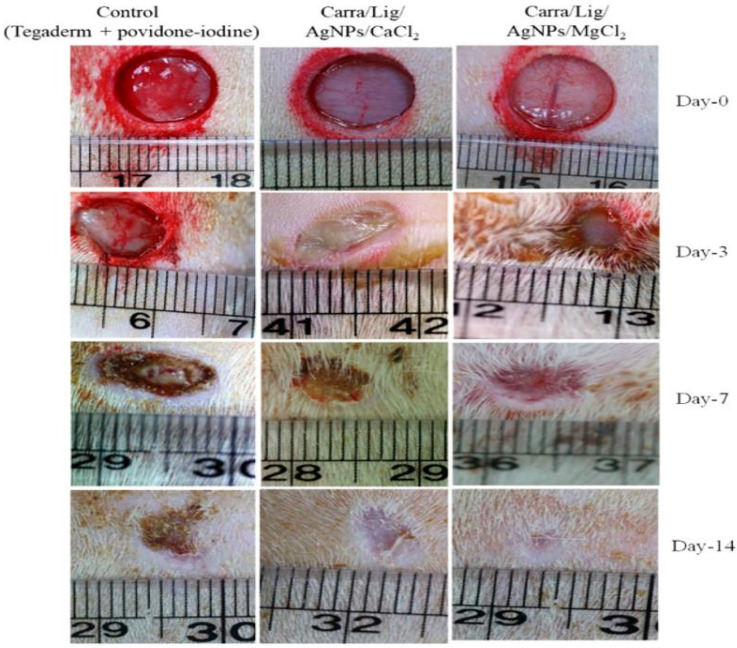
Wound healing effect of carrageenan-based nanocomposite hydrogels evaluated by the apparent photographs, which are showing wound healing progress. Reprinted with permission from [99], copyright 2020 Elsevier.

Jaiswal et al. [99] synthesized functional nanocomposite wound dressing materials based on carrageenan, using lignin as a reducing and capping agent in the carrageenan matrix, crosslinking metallic divalent cations such as CaCl_2_, CuCl_2_, and MgCl_2_. Green synthesis of silver nanoparticles (AgNPs) was prepared by the one-pot method. The crosslink density and physical connection between the polymer matrix and nanoparticles were increased as the nanocomposite hydrogels tensile strength increased. The physical properties, such as stability, swelling ratio, strength, and release rate of Ag ions, for carrageenan-based hydrogels containing AgNPs, have been improved by crosslinking with divalent cations depending on the type of crosslinking agent used. Carrageenan-based nanocomposite hydrogels have antimicrobial activity against *S. aureus* and *E. coli*. Carrageenan-based hydrogels are biocompatible with the fibroblast cell line of the mouse (L929 cell line). In Sprague Dawley rats, the Carra/Lig/Ag/MgCl_2_ hydrogel healed the wounds significantly within two weeks, reducing the wound region to less than 3%, further verified by the histological epidermis and mature gland examination (Figure 5). The carrageenan-based hydrogel has a high potential for wound dressing applications.

### 3.4. Titanium Dioxide Embedded in Wound Dressings

**Figure 6:**
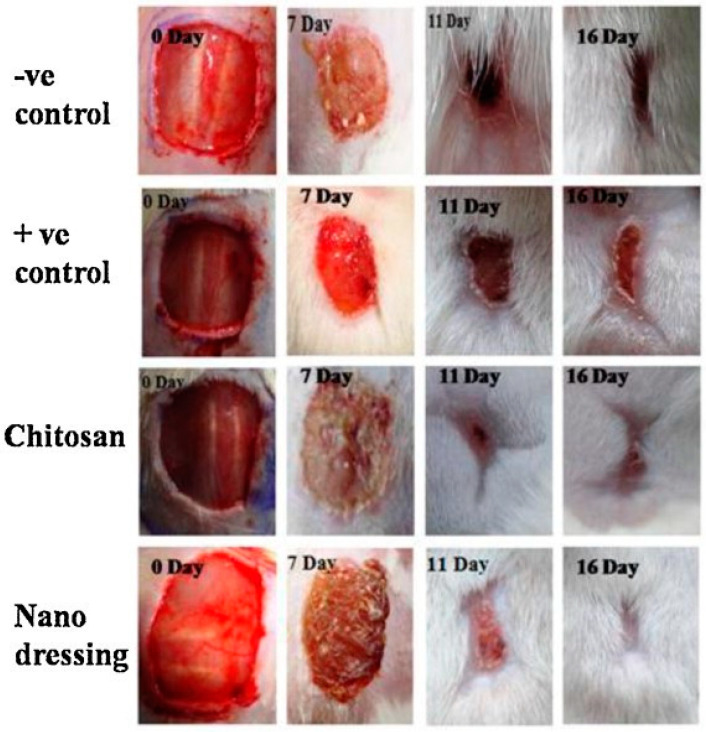
Appearances of wounds treated with controls, chitosan, and chitosan–PVP–TiO_2_ dressing. Reprinted with permission from [100], copyright 2013 Elsevier.

Archana et al. [100] prepared chitosan, poly(*N*-vinylpyrrolidone), and titanium dioxide (TiO_2_) blends by a casting method. The mechanical properties suggest that there is an increase in the strength of the composite dressing material by incorporating TiO_2_ nanoparticles. Nanocomposite materials have good antimicrobial properties and excellent biocompatibility against NIH3T3, and L929 fibroblast cells are contained in the prepared nanocomposite dressing. After 6 h, the dressing exhibits a maximum swelling rate of about 2289%. The WVTR exhibited by the Cs/PVP/TiO_2_ nano-dressing material is in the range of 1950 to 2050 g/m^2^/day, which is almost equal to the ideal value of a wound dressing. The zones of inhibition for Cs-PVP-TiO_2_ solution with four different pathogens are as follows: for *E. coli* it is 30 mm, *S. aureus* is 32 mm, *P. aeruginosa* is 38 mm, and *B. subtilis* is 28 mm. The negative control group of the wound closure rate at 3, 7, 11, and 16 days is 10.45%, 45.91%, 81.87%, and 90.42%, respectively. The positive control of the wound closure rate at 3, 7, 11, and 16 days is 27.79%, 55.58%, 86.51%, and 93.34%, respectively. The chitosan–PVP–TiO_2_ wound closure rate at 3, 7, 11 and 16 days is 31.48%, 62.33%, 91.49%, and 99.09%, respectively. On the 16th day of the study, the wound treated with the chitosan–PVP–TiO_2_ nanocomposite film showed the entire surface of the wound healed completely, almost 99%, and the wound was covered with new epithelium when treated with this nano-dressing (Figure 6). The prepared nano-dressing produced faster healing on the open-type wounds in the albino rat model as compared to the soframycin skin ointment, traditional gauze, and chitosan-treated class. Excellent antibacterial properties, high water vapor transmission rate, high swelling properties, good hydrophilic nature, biocompatibility, wound closure rate, and wound appearance makes chitosan–PVP–TiO_2_ nano-dressing a good candidate for wound healing applications.

Montaser et al. [101] fabricated a nanocomposite salicylamide/chitosan/TiO_2_ membrane via a casting technique. For the fabricated membranes, the SA/CS/TiO_2_ swelling percent is 1500%. The antibacterial activity of the crosslinking effect against *P. aeruginosa* and *S. aureus* was studied using CFU methods. Crosslinking was enhanced using SA and TiO_2_, until 1 mL of SA and 0.5 mg of TiO_2_ inside the membrane formulation was reached. The prepared membranes are fit to be used as antibacterial membranes, especially in wound dressing and food packing applications.

Archana et al. [102] prepared another nano-dressing membrane, containing chitosan–TiO_2_–pectin, using a solution casting method. Morphological evidence shows that TiO_2_ nanoparticles are well incorporated into the dressing material. Increasing the concentration of titanium dioxide nanoparticles and decreasing pectin content will enhance the tensile strength of the blend. Different methods, such as the hemolysis ratio measurement, whole-blood clotting test, cytotoxicity test using NIH3T3, and L929 fibroblast cells, are used for evaluating the physicochemical parameters of the nano-dressing. Measuring wound contraction and histological examinations in adult male albino rats helps to evaluate the in vivo open excision-type wound healing efficiency of the prepared nano-dressing compared with traditional gauze. The zones of inhibition for the CS–TiO_2_–pectin solution with four different pathogens are as follows: for *E. coli* it is 45 mm, *S. aureus* is 45 mm, *B. subtilis* is 49 mm, and *A. niger* is 29 mm. The chitosan–TiO_2_–pectin dressing material induced 1.14% of the contacting erythrocytes to hemolyze over 60 min of contact with whole blood. The gauze-treated group’s wound closure rate at 3, 7, 11, and 16 days was found to be 17.45%, 46.98%, 82.87%, and 91.22%, respectively. The chitosan-treated group’s wound closure rate at 3, 7, 11, and 16 days is 28.79%, 56.98%, 87.11%, and 94.98%, respectively. The wound closure rate of the chitosan–pectin–TiO_2_ dressing-treated group at 3, 7, 11, and 16 days is 32.98%, 63.43%, 92.45%, and 99.01%, respectively. On the 16th day of the study, the entire surface of the wound healed completely, almost 99%, and the wound covered with new epithelium when treated with the nano-dressing. Excellent antibacterial properties, high water vapor transmission rate, high swelling properties, and good hydrophilic nature, biocompatibility, wound closure rate, wound appearance, and histological analysis through in vivo testing suggest that the chitosan–pectin–TiO_2_ nano-dressing is a good candidate for wound healing applications.

Ulu et al. [103] prepared a novel chitosan (CH)/poly(propylene glycol) (PPG)/titanium dioxide (TiO_2_), and composite hydrogel films were processed by a solution casting method. TiO_2_ nanoparticles (NPs) had an average size distribution of around 40 nm. The CH/PPG film had an average thickness of 0.372 ± 0.008 mm, with a porosity of 95.4%, and a CH/PPG/TiO_2_ composite film thickness of about 0.343 ± 0.154 mm, and a porosity of about 94.6%. The water retention percentage for the CH/PPG film was over 77.3% after 60 min, while for the CH/PPG/TiO_2_ composite film it was 68.5%. The tensile strength of the CH/PPG film and CH/PPG/TiO_2_ film is 1.88 MPa and 3.0 MPa, respectively. The elongation breaks values of the CH/PPG film and CH/PPG/TiO_2_ composite films are 27% and 31%, respectively. Due to the addition of TiO_2_ nanoparticles, the contact angle decreased from 44.7° to 38.7°. The WVTR value of the CH/PPG films is about 365.0 g/m^2^/day, and for the CH/PPG/TiO_2_ composite films, it is 369.3 g/m^2^/day. The antimicrobial efficacy for the CH/PPG/TiO_2_ composite film caused a higher zone of inhibition against *E. coli*, *S. aureus*, and *C. lipolytica* compared to the CH/PPG film, which was examined by the disc diffusion method. The CH/PPG/TiO_2_ composite hydrogel film exhibits excellent antimicrobial activity. The prepared composite scaffold is used for biomedical applications.

### 3.5. Iron Oxides Embedded in Wound Dressings

Cai et al. [104] fabricated magnetic iron oxide nanoparticles (Fe_3_O_4_ NPs)/chitosan (CS)/gelatin (GE) nanofiber membranes by an electrospinning method. The highest mechanical strength was exhibited on 1 wt.% Fe_3_O_4_/CS/GE nanofiber membranes, with a 155% increase in Young’s modulus, 128% increase in tensile strength, and a 100% improvement in toughness from CS/GE. With the addition of Fe_3_O_4_ particles, the homogenous morphology was sustained. Moreover, the Fe_3_O_4_ concentration increased up to 4 wt.%, and the composite nanofiber diameter improved from 307 to 435 nm. The processed material shows a higher antibacterial assay against *S. aureus*, which is 20 mm, and *E. coli* is 21 mm, which improves the fabricated Fe_3_O_4_/CS/GE nanofiber membranes. Thus, Fe_3_O_4_/CS/GE membranes with optimized mechanical and antibacterial properties might be used in wound dressing applications.

Cao et al. [105] prepared citrate-modified maghemite-incorporated chitosan-coated porous cellulose membranes (MCM-CS) by the freeze–thaw method. Moreover, as the concentration of chitosan was raised, the tensile strength progressively improved. The tensile strength of MCM is 74.06 MPa, with MCM-CS2 at 3.07 MPa, MCM-CS3 at 100.36 MPa, and MCM-CS4 at 107.77 MPa. The swelling, BSA, and WVTR values for MCM-CS4 are 224.39%, 109.85 mg/g, and 879 g/m^2^/day, respectively. The antibacterial efficiency against *S. aureus* and *E. coli* was investigated. The zone of inhibition was measured for all of the samples. All the results prove MCM-CS as a potential applicant for wound dressing applications.

Sathiyaseelan et al. [106] prepared a polyvinyl alcohol (PVA)/CS-PD (*Pinus densiflora*)–iron oxide (FeO NPs) composite scaffold using the freeze drying method. However, the higher concentration of PD–FeO NP-incorporated sponges (0.03 and 0.05%) exhibited a decreased porosity when compared to CS/PVA–PD–FeO NPs (0.01%). In CS/PVA–PD–FeO NPs (0.01%), the maximum water absorption of 2855.55 ± 83.80% was found at 6 h. The antibacterial activity against *S. aureus*, *E. coli*, *B. cereus*, and *S. enterica* was assessed. The inhibition zone was detected in CS/PVA–PD–FeO NPs (0.01%) composite sponges, when tested against *S. aureus* (21 ± 1 mm), *E. coli* (20 ± 2 mm), *B. cereus* (22 ± 2 mm), and *S. enterica* (22 ± 1.5 mm). As a result, the treated samples do not show significant cytotoxicity in HEK-293 cells up to the tested concentration (1 mg/mL). CS/PVA–PD–FeO NPs (0.01%) augmented cell proliferation in HEK-293 cells observed in the in vitro wound healing scratch assay. The CS/PVA–PD–FeO NPs (0.01%) sponge would be endorsed for diabetic wound dressing after a detailed in vivo evaluation.

Ahmed et al. [107] synthesized the Ag–Fe_3_O_4_@PCL nanofibers by using the electrospinning method. Magnetite nanoparticles (Fe_3_O_4_ MNPs), doped with different concentrations of Ag ions, were synthesized using the co-precipitation method. Morphological investigations showed that the average size of the nanoparticle agglomerates decreased with the addition of Ag, due to induced crystallographic disorder, while Ra and Rp, as the measures of the surface roughness, increased from 42.7 to 170.9 nm for 0.0Ag-MNPs, to 61.1 and 276.7 nm for 0.2Ag-MNPs. The average roughness (Ra) showed a slight respective increase with the addition of silver, for 0.0Ag-MNPs@PCL it is 31.6 ± 6.5, 0.1Ag-MNPs@PCL is 32.0 ± 3.4, and 0.2Ag-MNPs@PCL is 34.0 ± 4.5 nm. The tensile strength and contact angle for 0.0Ag-MNPs@PCL are 4.15 ± 0.21 MPa and 105.4 ± 4.5°, 0.1Ag-MNPs@PCL is 3.93 ± 0.32 MPa and 96.3 ± 5.2°, and 0.2Ag-MNPs@PCL is 4.42 ± 0.25 MPa and 88.5 ± 4.1°, respectively. The 0.0Ag-MNPs@PCL did not show an initiatory zone for the pathogens, 0.1Ag-MNPs@PCL showed antibacterial activity, with the zone of inhibition being 79.2 ± 4.5 and 80.1 ± 4.9% against *E. coli* and *S. aureus*, respectively. For the 0.2Ag MNPs@PCL scaffolds, there is an increase in the zone of inhibition to 87.5 ± 5.7 and 84.3 ± 7.5%, for *E. coli* and *S. aureus*, respectively. In vivo tests were conducted on rats, the 0.2Ag-MNPs@PCL scaffolds exhibited an average wound healing rate of 92 ± 3%, which was the highest healing rate among all of the sample groups. The results suggest an excellent potential for these composite nanofibrous scaffolds as useful wound dressing materials.

### 3.6. Graphene Oxides Embedded in Wound Dressings

Chen et al. [108] incorporated polyhexamethylene guanidine (PHMG)-modified graphene oxide (mGO) into the poly (vinyl alcohol) (PVA)/chitosan (CS) films, and processed the samples by a solution casting method. The PVA/CS/0.5 wt.% mGO hydrogel tensile strength and Young’s modulus for the dry film are 60.32 ± 3.19 MPa and 46.32 ± 12.10 MPa, compared with swollen films at 9.32 ± 0.21 MPa and 15.63 ± 1.17 MPa, respectively. The average WVTRs of PVA/CS/mGO (0.1, 0.2, 0.5, and 1.0 wt.%) composite films are 721.76, 722.85, 671.25, and 718.18 g/m^2^/day, respectively. For the biocompatibility of the PVA/CS/mGO composite films, CCK-8 assays were applied to measure the viability of the HaCaT cells. In this study, the PVA/CS/mGO composite films with low concentrations of mGO (0.1, 0.2, 0.5 wt.%) revealed biocompatible surfaces that were suitable for further antibacterial experiments. As a result, greatly enhanced antibacterial efficiency against *S. aureus* and *E. coli* was shown, which could be the synergistic bactericidal effect of CS, GO, and PHMG. In vivo experiments demonstrated that PVA/CS/0.5 wt.% mGO displayed the best remarkably accelerated wound healing capabilities via enhancement of re-epithelialization. All of these results demonstrated the potential as a wound dressing application.

Rongxiu et al. [109] prepared SA (sodium alginate)/GO (graphene oxide)/PVA (polyvinyl alcohol) nanocomposite sponges by the freeze drying method. By increasing the GO content from 0% to 2%, the tensile strength and Young’s modulus of the sponges increased from 1.03 ± 0.15 to 1.91 ± 0.10 MPa, and from 1.21 ± 0.20 to 2.92 ± 0.11 MPa, respectively. All of the sponges exhibited excellent swelling abilities with an SR of higher than 1000%. A hemolysis assay was performed as an easy and reliable approach to evaluate the blood compatibility of materials. The HR value of SP is 1.22 ± 0.17%, SPG1 is 1.01 ± 0.07%, SPG2 is 0.72 ± 0.14%, and SPG3 is 0.26 ± 0.09%. The antibacterial efficiency against *S. aureus* and *E. coli* were tested with nanocomposite sponges. The antibacterial assay confirmed that the sponges, mainly SPG1, showed great potential as a wound-dressing material.

Lin et al. [110] prepared hydroxypropyl cellulose (H), chitosan (C), polyethylene oxide (P), and graphene (G) nanofiber membranes, processed by the electrospinning method. The tensile strength of the membranes was 1.38–1.82 MPa, with a swelling ratio up to 1330–1410%. The WVTR of the wet HCPG membrane was about 3100 g/m^2^/day, close to the recommended WVTR of wound dressings. HCP and HCPG nanofiber membranes were nontoxic to fibroblast cells. HCP and HCPG nanofiber membranes were tested against *E. coli*, resulting in 82.7 ± 3.9% and 44.8 ± 17.2%. Therefore, HCP and HCPG nanofiber membranes have the potential to become superior antibacterial wound dressings.

Sadeghianmaryan et al. [111] fabricated polyurethane (PU)/graphene oxide (GO) fibers by the electrospinning method. The diameters of the PU/2%GO electrospun fibers ranged from 20 to 180 nm, with a mean diameter of 105 nm. The tensile at break displayed a considerable increase after the addition of GO, and it reached a maximum value of 2.51 N. The water contact angle values of the wound dressing were decreased by increasing the content of GO. As the graphene oxide content in the sample was increased, the swelling ratio also increased. GO fibers have strong inhibition capability, and are more responsible for antibacterial and antifungal effects than PU. The average cell viability values were over 80%, which displays good biocompatibility properties. PU/GO fibers show good potential as wound dressing materials.

### 3.7. Mesoporous Silica and Carbon Nanotubes Embedded in Wound Dressings

Wang et al. [112] prepared mesoporous silica-supported *N*-halamine precursor (MSSNP) chitosan (CS) siloxane aerogels, which were processed by the freeze drying method. The contents of chitosan, mesoporous materials, and *N*-halamine polymers have influenced the hemolysis ratio slightly. Due to very little tissue fluid existing in the wound, the released chlorine level was safe to the human body, and the stable release property of aerogel10-Cl made the aerogel possible as a wound dressing. Aerogel–Si and aerogel–Zeo caused 45.45% and 52.73% reductions in *S. aureus*, and 30.48% and 62.85% reductions in *E. coli*, respectively. All of the results for the chitosan/mesoporous silica hybrid siloxane aerogels show potential for wound dressing applications.

Shen et al. [113] prepared a porous composite membrane composed of cellulose and mesoporous SBA-15, processed with the aid of the protection of PBS prefilled in an SBA15 mesoporous channel against the chemical erosion of SBA-15 in an alkali environment. The tensile strength and Young’s modulus of P-CM-SBA (30 wt.%) are 10.25 ± 0.92 MPa and 32.37 ± 3.49 MPa, respectively, and the swelling behavior of P-CM-SBA (30%) is 116.1%. The WVTR of P–CM–SBA (30%) is 978 g/m^2^/day. The inhibitory zone was recorded for *S. aureus* P–CM–SBA (30%) at 99.8 ± 0.1%, and for *E. coli* at 99.9 ± 0.1%. All of these results of P–CM–SBA (30%) show great promise as wound dressing materials.

Liao et al. [114] prepared a composite dressing of carbon nanotubes (CNTs)/polyvinyl alcohol (PVA)/epidermal growth factor (EGF), processed by the electrospinning method. The fractured non-woven fabrics were not observed at high power, and the size was ~500 nm. The bio-actively released EGF also accelerated the growth of L929 fibroblasts, which showed its potential application in wound dressing. The RGR of cells tested by the MTT assay was 117.6% in experimental group 1 and 112.5% in experimental group 2 after 24 h, then 137.3 and 125.7% after 48 h, respectively. The wound-healing rate on the 3rd day post-treatment in each group ranged between 15.225 ± 1.034 and 16.726 ± 1.634%, with no difference observed between the groups. At 7 and 10 days, the healing rate in the experimental group was 60.19 ± 3.837 and 95.033 ± 6.247%, respectively. The results showed that the in vitro and in vivo assays positively influenced the designed dressing materials. In summary, all the recently used polymers and nanoparticles in wound dressing, along with their corresponding synthetic methods, are listed in Table 2.

## 4. Conclusions

Biomaterials play a vital role in biomedical applications. Wound dressing biomaterials must have good antibacterial activity and mechanical strength, hemostatic properties, water vapor permeability, adequate exchange of gas capacity, and enhanced free radical scavenging. These properties, if properly applied, can improve wound healing. Furthermore, dressings should be non-toxic, biocompatible, non-allergic, bio-stable, biologically adhesive, biodegradable, and simple to remove after healing. Metal oxide nanoparticles enhance the antimicrobial activity of the biopolymers. This review is a study of the latest literature in the field of wound dressing, using biopolymer and synthetic polymer-based nanocomposites. When considering these products, the choice of wound dressing will make a major difference in how well wounds heal. All of the results show that nanocomposite dressings outperform unmodified polymers in terms of mechanical properties, biocompatibility, and healing rate. The papers discussed herein have proffered revolutionary ideas regarding the different types of biomaterials available for wound dressing applications.

## Figures and Tables

**Table 1:** Chemical structures, properties and biomedical application of the biopolymers and synthetic polymers.

Biopolymers/Synthetic Polymers	Chemical Structures	Properties	Biomedical Applications	Ref.
Chitin and Chitosan	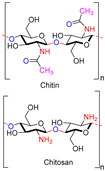	Non-toxic, biodegradable, biocompatible, antibacterial, anti-inflammatory	Wound dressing, drug delivery, bone regeneration, food packing, tissue engineering, cosmetics, etc.	[20,21,22,23,24]
Starch	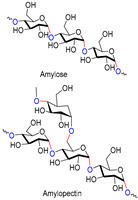	Biocompatible, non-toxic, biodegradable, non-carcinogenic, low immunogenicity	Wound dressing, artificial organs, drug delivery, tissue engineering, biosensor, etc.	[28,29,30]
Cellulose	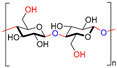	Biocompatible, biodegradable, non-toxic, high exudate ability, thermal stability	Artificial skin, blood vessels, drug delivery, tissue engineering, bone regeneration, etc.	[33,34,35,36]
Hyaluronic acid	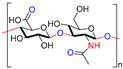	Non-toxic, biodegradable, biocompatible, non-immunogenicity, non-inflammatory	Wound healing, drug delivery, tissue engineering, cosmetics, etc.	[40,41,42]
Alginate	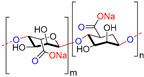	Non-toxic, biocompatible, biodegradable, bio-stable	Drug delivery, wound dressings, tissue engineering, blood vessels, bone regeneration, etc.	[47,48,49]
Poly(vinyl alcohol) (PVA)	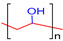	Non-toxic, biocompatible, biodegradable, mechanical strength, good hydrophilicity, thermal stability, excellent film forming	Wound dressing, tissue engineering, contact lenses, drug delivery, water filtration, implantable devices, food packaging, etc.	[55,56,57]
Poly(ethylene glycol) (PEG)	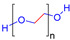	Non-toxic, bio-inert, non-immunogenic, biocompatible	Wound healing, tissue engineering, drug delivery, etc.	[52,58,59]
Poly (methacrylic acid) (PMAA)	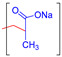	Biocompatible, non-toxic, high swelling capacity	Drug delivery, wound dressing, tissue engineering, etc.	[60,61,62,63]
Polyvinylpyrrolidone (PVP)	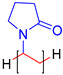	Non-toxic, biocompatible, excellent film forming	Tissue engineering, wound dressings, artificial pancreas, cardiovascular device, artificial skin, food packaging, etc.	[28,64,65]
Polycaprolactone (PCL)	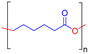	Biodegradable, biocompatibility, good mechanical properties	Wound dressing, tissue engineering, drug delivery systems, packaging, etc.	[66,67,68]
Poly (lactic acid) (PLA)	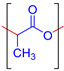	Biodegradability, biocompatibility, mechanical strength, thermal stability.	Tissue engineering, wound dressing, drug delivery, packaging, etc.	[69,70,71]
Polyurethane (PU)	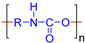	Non-toxic, biodegradable, excellent mechanical property, excellent film forming, adhesive.	Biosensor devices, drug delivery, wound dressing, tissue regeneration, etc.	[72,73]

**Table 2:** Summary of recently used biopolymers, synthetic polymers and nanoparticles in wound dressing.

Membrane Composition	Method of Preparation	Antibacterial Strain	Cell Line Used	Ref.
ZnO/MCM-41	Solution casting	*S. aureus* and *E. coli*	**-**	[81]
Heparinized ZnO/Poly(vinylalcohol)/Carboxymethyl Cellulose	Freeze–thaw	*S. aureus* and *E. coli*	L-929 and Human dermal fibroblasts	[82]
ZnO/Poly(vinyl alcohol)/Chitosan	Freeze–thaw	*S. aureus* and *E. coli*	Mouse fibroblast cells (L-929)	[83]
ZnO/Hyaluronic acid	One-pot synthesis	*S. aureus*, *B. subtilis*, *E. coli*, *P. aeruginosa*, and *V. cholerae*	Human skin Fibroblasts	[84]
Polyurethane/ZnAg	Electrospun	*E. coli*, *S. aureus* and *B. subtilis*	-	[85]
ZnO -coated silk fibroin fabric	-	*E. coli*	L929 Fibroblast cells	[86]
CeO_2_/Poly (ε-caprolactone)/Gelatin	Electrospun	-	L929 Murine fibroblast cell	[87]
CeO_2_/Peppermint oil on polyethylene oxide/Graphene oxide	Electrospun	*S. aureus* and *E. coli*	-	[88]
CeO_2_/Chitosan/cellulose acetate	Solution Casting	*S. aureus* and *E. coli*	-	[89]
Chitosan/Poly(vinyl alcohol)/CeO_2_	Freeze-thaw	*S. aureus* and *E. coli*	Human dermal fibroblast cells	[90]
Polycaprolactone/Ag	Electrospun	*S. aureus* and *E. coli*		[91]
Chitosan/Poly(vinyl lcohol)/Ag	Freeze dryer	*P. aeruginosa* and *S. aureus*	Fibroblast cells	[92]
Polylactide/Ag	Electrospun	*S. aureus* and *E. coli*	Fibroblast cells	[93]
Silkfibroin/chitin/Ag	Freeze dryer	*E. coli*, *S. aureus* and *C. albicans*	-	[94]
Polyurethane/lavender oil/Ag	Electrospun	*S. aureus* and *E. coli*	-	[95]
Ag/Eggshell membrane	Chemical reduction	*S. aureus* and *E. coli*	-	[96]
Chitosan/Poly(vinyl alcohol)/AgNO_3_ and vitamin E	Solution Casting	*Salmonella typhimurium*	-	[97]
Poly(vinyl alcohol)/Starch/g-C_3_N_4_/Ag@TiO_2_	Solution casting	*S. aureus* and *E. coli*	-	[98]
Carra/Lig/Ag (Carra/Lig/Ag/CaCl_2_, Carra/Lig/Ag/CuCl_2_ and Carra/Lig/Ag/MgCl_2_)	Solution Casting	*S. aureus* and *E. coli*	Mouse fibroblast cell line	[99]
Chitosan/poly(*N*-vinylpyrrolidone)/TiO_2_	Solution Casting	*E. coli*, *S. aureus*, *B. subtilis* and *P. aeruginosa*	NIH3T3 and L929 fibroblast cells	[100]
Salicylimine-Chitosan/TiO_2_	Solution Casting	*S. Aureus*, *P. aeruginosa*	**-**	[101]
Chitosan/pectin/TiO_2_	Solution Casting	*E. coli*, *S. aereus*, *P. aeruginosa*, *A. niger*, *B. subtilis*	NIH3T3 and L929 fibroblast cells	[102]
Chitosan/poly (propylene glycol)/TiO_2_	Solution Casting	*E. coli*, *S.aureus*, and *C.lipolytica*	**-**	[103]
Fe_3_O_4_/Chitosan/Gelatin	Electrospun	*S. aureus* and *E. coli*	-	[104]
Fe_2_O_3_, γ/Chitosan/porous cellulose membranes	Freeze dryer	*S. aureus* and *E. coli*	-	[105]
Chitosan/Poly(vinyl alcohol)/FeO	Freeze dryer	*S. aureus* and *E. coli*	HEK-293 cells	[106]
Ag doped Fe_3_O_4_/Poly caprolactone	Electrospun	*S. aureus* and *E. coli*	Human melanocyte cell line, HFB4	[107]
Poly(vinyl alcohol)/Chitosan/mGO	Solution casting	*S. aureus* and *E. coli*	Human keratinocyte cells	[108]
Sodium alginate/Graphene oxide/Poly(vinyl alcohol)	Freeze dryer	*S. aureus* and *E. coli*	NIH 3T3 cells	[109]
Hydroxypropyl cellulose/Chitosan/polyethylene oxide/graphene (HCPG)	Electrospun	*S. aureus* and *E. coli*	Human fetal skin fibroblast cells	[110]
Polyurethane/graphene oxide (GO)	Electrospun	*S. aureus* and *E. coli*	Human dermal fibroblast	[111]
Chitosan/Mesoporous silica	Freeze dryer	*S. aureus* and *E. coli*	-	[112]
Mesoporous silica/cellulose	Solution casting	*S. aureus* and *E. coli*	-	[113]
Carbon nanotubes/Poly(vinyl alcohol)/epidermal growth factor (EGF)	Electrospun	-	L929 mouse fibroblast	[114]

## Data Availability

The data presented in this study are available in the below listed references.
